# Potential Mechanisms and Effects of Dai Bai Jie Ethanol Extract in Preventing Acute Alcoholic Liver Injury

**DOI:** 10.3390/cimb47010003

**Published:** 2024-12-24

**Authors:** Niantong Xia, Hongwei Xue, Yihang Li, Jia Liu, Yang Lou, Shuyang Li, Yutian Wang, Juan Lu, Xi Chen

**Affiliations:** 1 State Key Laboratory for Quality Ensurance and Sustainable Use of Dao Di Herbs, Institute of Medicinal Plant Development, Chinese Academy of Medical Sciences, Peking Union Medical College, Beijing 100193, China; 18263825319@163.com (N.X.); 15297318065@163.com (H.X.); lj200929@126.com (J.L.); louyang0727@163.com (Y.L.); 15563999708@163.com (S.L.); wytqingya@163.com (Y.W.); 2 Yunnan Branch, Institute of Medicinal Plant Development, Chinese Academy of Medical Sciences, Jinghong 666100, China; yhli@implad.ac.cn

**Keywords:** Dai Bai Jie, AALI, saponin, NF-κB, ADH/ALDH

## Abstract

This study investigated the protective effect of Dai Bai Jie (DBJ) extract against acute alcoholic liver injury (AALI) and elucidated its potential mechanism. The total saponin level in the DBJ extracts was measured using vanillin–chloroform acid colorimetry. To observe the preventive and protective effects of DBJ on AML-12 cells in an ethanol environment, the effective components of DBJ were identified. An alcohol-induced AALI mouse model was used to evaluate the efficacy of DBJ against AALI. For this purpose, alcohol dehydrogenase (ADH) and acetaldehyde dehydrogenase (ALDH) levels were assessed, liver function indices and oxidative and inflammatory markers were determined, and histopathological examinations were performed. Mechanistic investigations were conducted using RT-qPCR assays and immunohistochemical analysis to determine the protective effects of DBJ. The samples (DBJ-1, DBJ-2, and DBJ-3) were obtained by extracting DBJ with water, 50% ethanol, and 95% ethanol, yielding total saponin contents of 5.35%, 6.64%, and 11.83%, respectively. DBJ-3 was isolated and purified, and its components were identified by Ultra Performance Liquid Chromatography-Mass Spectrometry (UPLC-MS). DBJ-3 had the greatest effect on cell viability in an ethanol environment. Moreover, DBJ-3 reduced inflammatory infiltration, liver cell degeneration, and hemorrhage, while increasing ADH and ALDH levels in liver tissues. Additionally, DBJ-3 considerably decreased the serum alanine aminotransferase (ALT), aspartate aminotransferase (AST), total cholesterol (TC), and triglyceride (TG) levels. DBJ-3 reduced malondialdehyde (MDA), reactive oxygen species (ROS), and inflammatory factors, such as tumor necrosis factor (TNF-α), interleukin-1β (IL-1β), and interleukin 6 (IL-6), while increasing superoxide dismutase (SOD) and glutathione S-transferase (GST) activities. Furthermore, DBJ-3 significantly increased alcohol dehydrogenase 1b (ADH1B) and aldehyde dehydrogenase 2 (ALDH2) expression at the gene and protein levels within alcohol metabolism pathways and reduced the nuclear factor kappa-B (NF-κB) gene and protein levels. These findings suggest that DBJ-3 can prevent AALI by enhancing alcohol metabolism via the regulation of ADH1B and ALDH2 and the modulation of the NF-κB pathway to improve antioxidant and anti-inflammatory effects.

## 1. Introduction

Acute alcoholic liver injury (AALI) is regarded as a significant contributor to deaths in individuals who engage in alcohol abuse [1,2]. As alcohol consumption continues to rise, the associated health challenges posed by AALI are escalating [3]. The liver is essential for human metabolism and is susceptible to damage from excessive alcohol intake. Upon ingestion, alcohol is absorbed via the gastrointestinal tract and predominantly metabolized into acetaldehyde by ADH, which is subsequently converted into benign substances by ALDH [4,5]. Acute or short-term excessive alcohol intake can overwhelm the liver’s metabolic capacity, leading to the accumulation of toxic substances, such as acetaldehyde, which adversely affect both the body and the liver [6,7]. Enzymatic activity related to ethanol metabolism in the body influences alcohol tolerance and susceptibility to AALI [8,9]. ADH and ALDH play pivotal roles in the onset of AALI [10,11]. The pathogenesis of AALI involves ethanol and acetaldehyde toxicity to the liver, lipid peroxidation, the generation of reactive oxygen species (ROS) (the general term for peroxides, which are related to oxygen metabolism, oxygen-containing free radicals and easily form free radicals in living organisms), oxidative stress, cytokine- and chemokine-mediated inflammatory responses, liver metabolic disturbances, regeneration, and bacterial translocation following alcohol consumption [12,13,14].

Moreover, AALI can progress from early alcoholic fatty liver (AFL) to alcoholic steatohepatitis (ASH), with chronic ASH potentially leading to liver fibrosis, cirrhosis, and hepatocellular carcinoma (HCC), either independently or simultaneously. Liver fibrosis and cirrhosis resulting from ASH are irreversible, highlighting the need for early intervention in AALI [15]. Current treatment strategies for AALI primarily emphasize alcohol abstinence along with nutritional and pharmacological interventions. While these approaches aim to mitigate the severity of alcoholic liver disease and address secondary malnutrition, they do not fully reverse alcoholic liver injury. Pharmacological treatments often exhibit limited efficacy, poor patient compliance, and the potential for adverse reactions and toxic side effects. For example, although corticosteroids are used to manage alcoholic hepatitis, they offer temporary relief with varied patient responsiveness [16,17,18]. Similarly, glucocorticoids, alone or in combination with other drugs, can treat AALI but are associated with significant side effects or incomplete recovery [19], limiting their suitability for long-term use [20]. Drugs such as acamprosate, disulfiram, and naltrexone, which are proposed for AALI treatment, have not gained widespread international acceptance [21]. Currently, no safe and effective medication exists for acute alcohol-induced liver injury, necessitating the development of drugs for preventing and treating all stages of AALI. Several studies have suggested that natural plant components such as polysaccharides [22], saponins [23], polyphenols [24], and flavonoids [25,26] exhibit promising protective effects against AALI. These substances are environmentally friendly and have minimal toxic side effects, potentially offering safe and effective treatments for AALI.

As one of four major ethnic medicines in China, Dai medicine plays a crucial role in healthcare in China and parts of Southeast Asia [27]. The principles and practices of toxicity resolution based on Dai medicine are fundamental to disease prevention and treatment [28]. Dai physicians have long applied the principle of “resolving before illness, treating after resolution, and simultaneous treatment during resolution” in their medical practice [29,30]. DBJ, a traditional Dai herb also known as “Ya Jie Xian Da” in the Dai language, translates to “medicine for resolving hundreds of poisons”. It is derived from the dried root of *Marsdenia tenacissima* Moon from the Asclepiadaceae family. In folk medicine, DBJ is widely used for detoxifying alcohol and drugs. It is a key ingredient in several nationally approved formulations, such as Ya Jie Pian, and various preparations used in Dai hospitals, such as Bangla Liquid, Bai Jie Capsules, and Ya Jie Gahan. Despite its recognized therapeutic effects in folk medicine, further scientific validation and research are necessary.

To elucidate the mechanism through which DBJ mitigates alcohol-induced liver damage, in this study, we used different polar solvents for DBJ extraction. The components of the DBJ extract were identified by UPLC-MS. AML-12 cells were used to simulate alcohol-induced injury, and the active components of DBJ were identified. Furthermore, a mouse model of AALI was developed to investigate the pharmacological effects and potential mechanisms of DBJ.

## 2. Materials and Methods

### 2.1. Materials and Chemicals

Dai Bai Jie was purchased from Lin Yanfang Dai Medicine, Hall Gasa Clinic in Xishuangbanna. Er Guo Tou liquor (containing 56% ethanol) was purchased from Beijing Red Star Co., Ltd. (Beijing, China). Vanillin was purchased from Tianjin Fuchen Chemical Reagent Factory (Tianjin, China), glacial acetic acid from Tianjin Yongda Chemical Reagent Co., Ltd. (Tianjin, China), and perchloric acid from Tianjin Xinyuan Chemical Co., Ltd. (Tianjin, China). Total saponin content Kit (the standard product is ginsenoside Re) was purchased from Nanjing JC DTECT Biotechnologies Co., Ltd. (Nanjing, China). The H&E staining solution, differentiation solution, and counterstaining solution were purchased from Wuhan Servicebio Technology Co., Ltd. (Wuhan, China). Methanol, acetonitrile, and formic acid were of chromatographic grade. The 10x phosphate-buffered saline was purchased from Shanghai Jingxin Industrial Development Co., Ltd. (Shanghai, China). ADH, and ALDH activity detection kits were provided by Beijing Solarbio Science & Technology Co., Ltd. (Beijing, China). ADH, ALDH, ALT, AST, TG, LDH, TC, LDL-C, MDA, ROS, SOD, GST, CAT, IL-1β, IL-6, and TNF-α ELISA kits were purchased from Shanghai Enzyme-linked Biotechnology Co., Ltd. (Shanghai, China). Primers for ADH1B, ALDH2, Nrf2, HO-1, NF-κB, and TNF-α were purchased from Beijing Boao Sen Biotechnology Co., Ltd. (Beijing, China). The MCO-15AC CO2 incubator was acquired from SANYO DENKI Co., Ltd. (Osaka, Japan).The IX51 Leica DMIRB was obtained from Olympus Optical Co., Ltd. (Tokyo, Japan). The Nanodrop 2000 microvolume spectrophotometer was purchased from Thermo Fisher Scientific (Massachusetts, USA). The RNAprep FastPure Total RNA Extraction Kit (Double Column), TSINGKE TSK314M SynScript^®^III RT SuperMix for qPCR (+gDNA Remover), ArtiCanATM SYBR qPCR Mix, RNase-free water, and solid phase RNase scavenger were purchased from Beijing Qingke Biotechnology Co., Ltd. (Beijing, China). The TS-GelRed nucleic acid gel stain Ver.2 (10,000× aqueous solution) was sourced from Beijing Qingke Biotechnology Co., Ltd. (Beijing, China). The PCR transparent sealing film was purchased from Axygen (silicon valley, USA), and the DAB color development kit was acquired from Wuhan Servicebio Technology Co., Ltd. (Wuhan, China). The HRP-labeled goat anti-rabbit IgG and rabbit anti-ADH1B, ALDH2, Nrf2 (BS-1074R), anti-NF-κB p65 (BS-0465R), and anti-HO-1 (BS-2075R) antibodies were purchased from Wuhan Servicebio Technology Co., Ltd. (Wuhan, China).

### 2.2. Preparation of Extract from DBJ

To prepare the extracts, three portions of 100 g of Daibai cleaning powder were taken, and 1000 mL of distilled water, a 50% ethanol solution, or a 95% ethanol solution was added to each portion, maintaining a liquid-to-material ratio of 1:10. The three mixtures were stirred thoroughly and then subjected to reflux extraction three times, with each reflux lasting for 2 h. After filtration, the mixtures were filtered, and the filtrates were combined. The combined filtrate was concentrated using rotary evaporation and subsequently dried at 60 °C to yield three extracts, designated DBJ-1, DBJ-2, and DBJ-3.

### 2.3. Determination of Total Saponin Content in the Extract of DBJ

#### 2.3.1. The Vanillin–Perchloric Acid Colorimetric Method

The total saponin content was determined using the vanillin–perchloric acid colorimetric method [31,32]. A 5% solution of vanillin in acetic acid was prepared. The test solution (500 μL) was accurately transferred and dried in a 70 °C water bath. A solution containing vanillin in acetic acid (200 μL) and perchloric acid (800 μL) was subsequently added. The resulting mixture was heated for 20 min in a 55 °C water bath before being cooled to room temperature. Next, the test solution (200 μL) was precisely transferred to a 1 mL centrifuge tube and mixed with acetic acid (1 mL). Finally, the absorbance values were measured at 589 nm.

#### 2.3.2. Standard Curve Generation

Initially, 10.80 mg of the ginsenoside Re standard substance was weighed and placed into a 10 mL volumetric flask. Methanol was added to the flask up to the mark to prepare the reference solution. Next, 0.1, 0.2, 0.3, 0.4, 0.5, and 0.6 mL aliquots from the ginsenoside Re reference solutions were precisely transferred into separate 1 mL volumetric flasks [33,34,35]. A solution of ginsenoside Re at different concentrations was produced by adding methanol to the volumetric flasks and diluting it to 1 mL. The colorimetric analysis was performed as described in Section 2.3.1, and the absorbance was measured. The standard curve was plotted to obtain the regression equation.

#### 2.3.3. Sample Testing

About 100 mg of DBJ-1, DBJ-2, or DBJ-3 was weighed, placed in separate 10 mL volumetric flasks, and then diluted with anhydrous ethanol. Colorimetric analysis was performed as described in Section 2.3.1, the absorbance of the saponins was measured, and the total saponin yield was calculated using the regression equation obtained from the previous section.

(1) $$Total\;saponin\;content\left(\%\right)=\frac{C\times V}{m}\times 100\%,$$

C: The concentration of total saponins in the extract of DBJ (mg/mL);
  V: Extract volume of DBJ (in mL);
  m: The quality of the extract from DBJ (in mg).

### 2.4. Qualitative Analysis of DBJ-3 by UPLC-MS

#### 2.4.1. Dry Sample Extraction

Using vacuum freeze-drying technology, the biological samples were placed in a lyophilizer (Scientz-100F, NINGBO SCIENTZ BIOTECHNOLOGY CO., LTD, Zhejiang, China), and then the samples were ground (30 Hz, 1.5 min) to powder form by using a grinder (MM 400, RETSCH GmbH, Heidenheim, Germany). Next, 50 mg of sample powder was weighed via an electronic balance (MS105DM, Mettler-Toledo International, Inc, Delaware, USA), and 1200 μL of 70% methanolic aqueous internal standard extract precooled at −20 °C was added (less than 50 mg was added at a rate of 1200 μL of extractant per 50 mg of sample). The mixture was vortexed once every 30 min for 30 sec, for a total of 6 times. After centrifugation (10 revolutions at 12,000 rpm for 3 min), the supernatant was aspirated, and the sample was filtered through a microporous membrane (0.22 μm pore size) and stored in an injection vial for UPLC-MS analysis.

#### 2.4.2. UPLC Conditions

The sample extracts were analyzed using a UPLC-ESI-MS system and a tandem mass spectrometry system (AB Sciex Pte. Ltd., Framingham, MA, USA). The analytical conditions were as follows: UPLC: column, Agilent SB-C18 (1.8 µm, 2.1 mm * 100 mm; Agilent Technologies Inc., Palo Alto, CA, USA). The mobile phase consisted of solvent A, pure water with 0.1% formic acid, and solvent B, acetonitrile with 0.1% formic acid. Sample measurements were performed with a gradient program that employed the starting conditions of 95% A, 5% B. Within 9 min, a linear gradient to 5% A, 95% B was programmed, and a composition of 5% A, 95% B was maintained for 1 min. Subsequently, a composition of 95% A, 5.0% B was adjusted within 1.1 min and maintained for 2.9 min. The flow velocity was set as 0.35 mL per minute. The column oven was set to 40 °C, and the injection volume was 2 μL. The effluent was alternatively connected to an ESI-triple quadrupole-linear ion trap (QTRAP)-MS.

#### 2.4.3. Mass Spectrometry Conditions

The ESI source operation parameters were as follows: source temperature, 500 °C; ion spray voltage (IS), 5500 V (positive ion mode)/–4500 V (negative ion mode); ion source gas I (GSI), gas II (GSII), and curtain gas (CUR) were set at 50, 60, and 25 psi, respectively; and collision-activated dissociation (CAD) was high. The collision gas, nitrogen, was set to a medium level, and DBJ-3 was analyzed accurately and qualitatively via the MRM monitoring mode. DP (declustering potential) and CE (collision energy) for individual MRM transitions were performed with further DP and CE optimization. A specific set of MRM transitions was monitored for each period according to the metabolites eluted within this period. The software Analyst 1.6.3 was used to process the mass spectrum data and perform qualitative analysis via a standard database (MetwareBio Co., Ltd., Wuhan, China). The total ion current (TIC) of the sample, which represents the cumulative intensities of all ions present in the mass spectrum at each time point relative to time, is documented. In the accompanying figure, the abscissa denotes the retention time (Rt) for metabolite detection, whereas the ordinate indicates the ion current intensity of the detected ions, measured in counts per second (cps).

### 2.5. Cells and Experimental Design

#### 2.5.1. Cell Culture

AML-12 cells [36,37] were obtained from the Cell Resource Center of the Institute of Basic Medical Sciences, Chinese Academy of Medical Sciences. The cells were cultured in high-glucose DMEM/F12 medium supplemented with 5% horse serum (HS), 2.5% fetal bovine serum (FBS), 1% penicillin/streptomycin, and 1% nonessential amino acids (NEAAs) at 37 °C in a 5% CO_2_ incubator, with regular replacement of the medium [38,39,40]. Upon reaching 80% confluence, the cells formed a monolayer, and the medium was aspirated. The cells were washed three times with 10 mL of PBS to remove metabolites and dead cells. The mixture was subsequently digested by adding 0.25% trypsin (5 mL), adjusted based on the cell status, and this process was stopped with 5 mL of fresh medium. After gentle pipetting to ensure a uniform cell suspension, the cells were centrifuged at 1000 rpm for 5 min. The supernatant was discarded, and the cells were resuspended in 4 mL of fresh medium to obtain a single-cell suspension. The cells were then passaged into T75 culture flasks at a 4:1 ratio and incubated at 37 °C with 5% CO_2_. After the log-phase, healthy AML-12 cells were rinsed with PBS, and the cells were digested with 0.25% trypsin for 1 min. Digestion was stopped with complete medium, and then, AML-12 single-cell suspensions were prepared, counted using a hemocytometer, inoculated in 96-well plates at a density of 1 × 10^4^ cells per well, and incubated for 24 h.

#### 2.5.2. Cytotoxicity of the DBJ Extract

Cell culture was performed as described in Section 2.5.1, and the cells were washed with PBS. After 24 h of culture in serum-free medium containing DBJ-1, DBJ-2, and DBJ-3 at 16, 32, 64, 128, and 256 μg/mL, with six replicates per group, the original medium was aspirated, and 10% CCK-8 reagent (100 μL) was added. Next, the cells were incubated for 2 h at 37 °C in a 5% CO_2_ incubator. The absorbance values were recorded using a microplate reader at 450 nm, and Equation (1) was used to calculate cell viability [41,42,43].

This is Example 1 of an equation:

(2) $$\begin{array}{c} Cell\;viability=\\ \frac{Treatment\;group\;OD\;value-Blank\;group\;OD\;value}{Model\;group\;OD\;value-Blank\;group\;OD\;value}\times 100\%. \end{array}$$

#### 2.5.3. Ethanol-Induced Cytotoxicity Assay in AML-12 Cells

Cell culture was performed as described in Section 2.4.1, followed by a single wash with PBS. After 24 h [36,44,45,46,47] of incubation in serum-free medium supplemented with ethanol (0, 25, 50, 100, 200, and 400 mmol/L), with six replicates per concentration, the previous medium was replaced with 10% CCK-8 reagent (100 μL). The cells were then incubated for 2 h at 37 °C in an incubator containing 5% CO_2_. The absorbance readings were taken at 450 nm using a microplate reader, and the cell viability was calculated using Equation (1). During the ethanol exposure period, water containing ethanol at a concentration of 400 mmol/l was added to the incubator to prevent ethanol volatilization [47].

#### 2.5.4. Effect of 100 mmol/L DBJ Extract on the Viability of AML-12 Cells

Cell culture was performed following the methodology described in Section 2.4.1. After rinsing with PBS, the cells were cultured in serum-free medium supplemented with DBJ-1, DBJ-2, or DBJ-3 at concentrations of 32, 64, or 128 μg/mL, respectively. Following incubation for 2 h, the cells were exposed to serum-free medium containing ethanol to achieve a final concentration of 100 mmol/L. After 24 h of coculture, the medium was replaced with 10% CCK-8 solution (100 μL). The cells were incubated for 2 h at 37 °C in an atmosphere containing 5% CO_2_. Next, the absorbance was measured at 450 nm, and the cell viability was determined using Equation (1). During the ethanol exposure period, water containing the same concentration of ethanol was added to the incubator to prevent ethanol volatilization.

### 2.6. Animals and Experimental Design

#### 2.6.1. Animals

We obtained six-week-old male SPF-grade C57BL/6J mice from Beijing Huafukang Bioscience Co., Ltd. (Beijing, China) and Sibeifu (Beijing) Biotechnology Co., Ltd.(Beijing, China), with the health certificate number SLXD-20230330021 for the experimental animals. All animal experiments were conducted following the guidelines of the National Institutes of Health. Our study protocols were approved by the Animal Ethics Committee of the Institute of Medicinal Plant Development, Chinese Academy of Medical Sciences. The mice were acclimatized for one week before the formal experiment under housing conditions maintained at 23 ± 2 °C, 55 ± 5% relative humidity, and a 12 h/12 h light/dark cycle.

#### 2.6.2. Establishment of an AALI Mouse Model

Six-week-old male C57BL/6J mice (n = 60, weight: 18–22 g) were randomly allocated into six groups (n = 10): normal control (NC, distilled water), model (M, 56% Hongxing Erguotou at 15 mL/kg), silymarin (S, 60 mg/kg), and three DBJ-3 groups (DBJ-3-L, DBJ-3-M, and DBJ-3-H, 100, 300, and 600 mg/kg, respectively). Treatments were administered using gavage.

On the 18th day of continuous DBJ-3 or saline administration, 1 h after dosing, the NC group received saline at 15 mL/kg body weight, whereas the other groups received alcohol gavage at the same volume for four days. After 21 days, the mice were starved for 6 h with water ad libitum before being anesthetized. Blood samples (0.8–1.0 mL) were collected using orbital puncture, followed by centrifugation at 5000 rpm for 15 min at low temperature. The supernatants were stored at −80 °C.

An abdominal midline incision was made to access the liver for organ index calculation. Liver tissues were processed for histopathological analysis, and another portion was stored at −80 °C for subsequent experimental analysis. A total of 20 mg of liver tissue was combined with 200 mL of 10x PBS in a 1.5 mL centrifuge tube. The tube was placed in a grinder and ground at low temperature. The grinding frequency was set to 60 Hz, and the grinding duration was set to 60 s. After centrifugation at 2400× *g* and 4 °C for 15 min, the supernatants were collected for biochemical analysis. The remaining liver tissue was used for gene and protein level analysis.

### 2.7. Regulation of Serum Biochemical Indices in AALI Mice by DBJ-3

The serum AST, ALT, LDH, TC, TG, and LDL-C levels were measured according to the corresponding assay kit protocols [48,49]. To determine the mouse indicator content in each sample, the absorbance (optical density, OD) was measured at a wavelength of 450 nm using a microplate reader. The standard curve generated from the standards provided in the kit was used for accurate quantification.

### 2.8. Determination of Alcohol-Metabolizing Enzymes in the Mouse Liver

The levels of ADH and ALDH in liver tissue homogenates were analyzed following specific assay kit protocols. To determine the mouse indicator content in each sample, the absorbance (optical density, OD) was measured at a wavelength of 450 nm using a microplate reader. The standard curve generated from the standards provided in the kit was used for accurate quantification.

### 2.9. Histopathological Analyses of Liver Tissue

Liver tissues from each group of C57BL/6J mice were fixed in 10% neutral buffered formalin. After gradient ethanol dehydration and embedding in paraffin, 3–5 μm thick sections were prepared. These sections underwent sequential treatments: xylene I and II for 20 min each, absolute ethanol I and II for 5 min each, and then 75% ethanol for 5 min. After rinsing with distilled water, the sections were stained with hematoxylin for 3–5 min, followed by rinsing in distilled water. Differentiation was performed using a differentiation solution, followed by another rinse, bluing in a bluing solution, and a final wash with distilled water. The sections were subsequently dehydrated in 85% and 95% ethanol for 5 min each, followed by eosin staining for 5 min. Finally, the sections were sequentially treated with absolute ethanol I, II, and III, followed by xylene I and II for 5 min each, before they were mounted with neutral balsam. Pathological assessment of liver tissue damage was conducted under a microscope (Biological Microscope ECLIPSE E100, Nikon Instruments, Inc., Tokyo, Japan).

### 2.10. Liver Oxidative Stress Analysis

The ROS, SOD, MDA, CAT, and GSH levels in liver tissue homogenates were measured using ELISA kits. To determine the mouse indicator content in each sample, the absorbance (optical density, OD) was measured at a wavelength of 450 nm using a microplate reader. The standard curve generated from the standards provided in the kit was used for accurate quantification.

### 2.11. Inflammation Analysis

The TNF-α, IL-6, and IL-1β levels in the liver tissue homogenates were analyzed using ELISA kits. To determine the mouse indicator content in each sample, the absorbance (optical density, OD) was measured at a wavelength of 450 nm using a microplate reader. The standard curve generated from the standards provided in the kit was used for accurate quantification.

### 2.12. Gene Levels

1. The centrifuge column approach was used to extract total RNA from 20 mg of liver tissue.
2. The purity of the isolated RNA was assessed using a Nanodrop 2000 spectrophotometer, with an OD 260/280 ratio of approximately 2.0, suggesting high purity.
3. The RNA was treated with the kit reagents by incubation for 2 min at 42 °C and then for 1 min at 60 °C to remove residual genomic DNA. The mixture was subsequently incubated for 15 min at 50 °C and for 5 s at 85 °C to synthesize DNA.
4. An ABI Quantstudio 6 Flex real-time PCR system and an ArtiCanTM SYBR qPCR kit were used for quantitative polymerase chain reaction (qPCR). Specific primers were used for qPCR analysis. The cycling procedure included 1 min of initial denaturation at 95 °C; 40 cycles of 10 s of denaturation at 95 °C, 20 s of annealing at 60 °C, and 1 min of extension at 72 °C. *Gapdh* served as an internal reference. The 2^−ΔΔCt^ method was used to determine relative gene expression according to cycle threshold (Ct) values [50].

With *Gapdh* [51,52,53,54] as a reference gene, information on specific primers for the target genes *Adh1b*, *Aldh2*, *Nf-κb*, *Tnf-α*, and *IL-1β* are listed in Table 1.

### 2.13. Immunohistochemical Analysis

To assess the protein levels of ADH1B, ALDH2, and NF-κB, 4 µm sections were prepared from paraffin-embedded liver tissue and subjected to deparaffinization and hydration [55,56]. The sections were placed in a 10 μmol/L citrate buffer and subjected to antigen retrieval using microwave heating for 15 min, followed by cooling to room temperature. After rinsing three times with PBS (5 min per wash), excess liquid was removed by blotting with absorbent paper. Next, the sections were treated with peroxidase-blocking solution (50 μL) at room temperature for 20 min to inhibit endogenous peroxidase activity. The samples were then washed three times with PBS (5 min per wash), and excess liquid was removed using absorbent paper. Serum was added to the sections to block nonspecific binding sites for 30 min at room temperature. Following serum removal, primary antibodies, including those against ADH1B, ALDH2, and NF-κB, were added to the sections at 4 °C overnight. The sections were then rinsed three times with PBS (5 min each) before being incubated for an additional 50 min with horseradish peroxidase-labeled secondary antibodies at room temperature. Finally, DAB chromogen was added to the stained sections, followed by hematoxylin counterstaining to verify the staining intensity, dehydration, and mounting. A Nikon E100 optical microscope (Nikon Corporation, Tokyo, Japan) was used to observe the stained sections, and ImageJ 1.53t software (Rawak Software Inc., Stuttgart, Germany) was used for analysis.

### 2.14. Statistical Analysis

The experimental results were analyzed using one-way ANOVA in SPSS 28.0, and between-group multiple comparisons were performed using the least significant difference approach. The data are presented as the means ± standard deviations. All the results were considered statistically significant at *p* < 0.05. GraphPad Prism 10.0 software was used to generate graphs and charts.

## 3. Results

### 3.1. Determination of Total Saponin Contents in Different Extracts of DBJ

**Figure 1:**
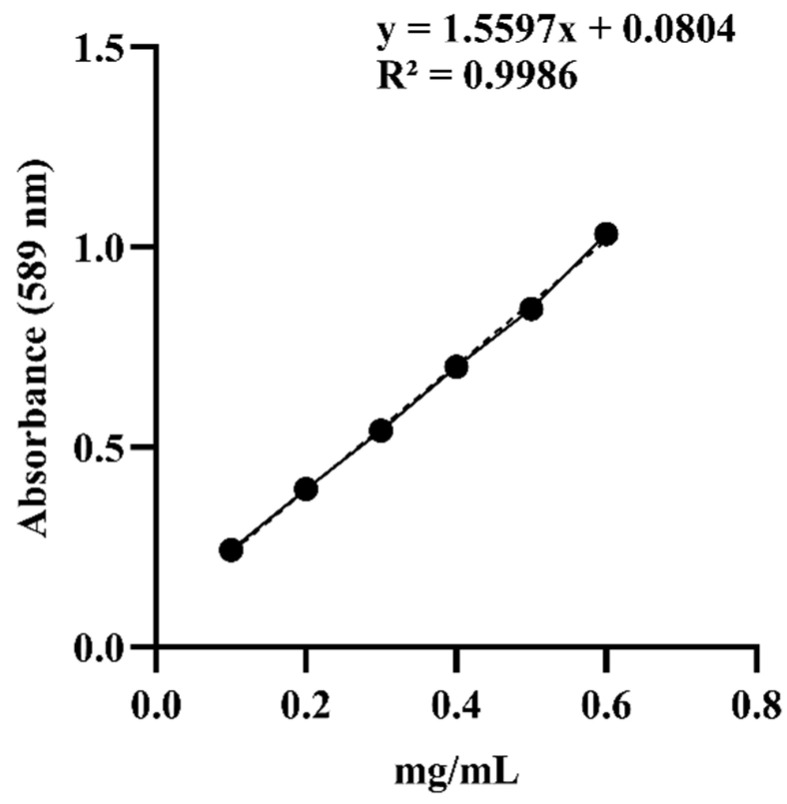
Standard curve of ginsenoside Re.

The vanillin–perchloric acid colorimetric method was used to measure the absorbance of ginsenoside Re standard solutions at different concentrations (Table 2), and a standard curve was constructed (Figure 1). The regression equation correlating the ginsenoside Re concentration with the absorbance was y = 1.559x + 0.0926, and the correlation coefficient (R^2^) was 0.9991, indicating a robust linear relationship. Based on these calculations, the total ginsenoside contents of DBJ-1, DBJ-2, and DBJ-3 were determined to be 5.35%, 6.64%, and 11.83%, respectively.

### 3.2. Phytochemical Analysis of DBJ-3

**Figure 2:**
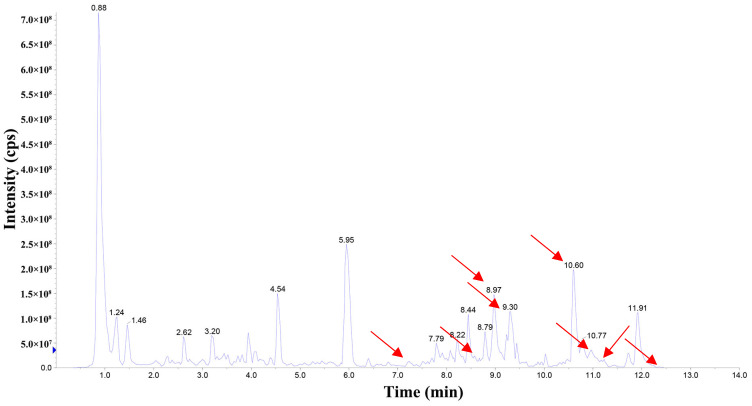
Total ion chromatogram of DBJ-3 in negative ion mode. The arrow in Figure 2 indicates the detected saponin.

The components of DBJ-3 were analyzed via UPLC-MS, resulting in the identification of a total of eight known saponin structures. The structures, retention times (tR), and formulas of these saponins are detailed in Table 3. Additionally, the total ion chromatograms (TICs) are shown in Figure 2.

### 3.3. Establishment of an Ethanol-Mediated AML-12 Cell Injury Model and Screening of the Effective Fractions of DBJ

#### 3.3.1. Cytotoxicity Evaluation of DBJ Extract on Cells

**Figure 3:**
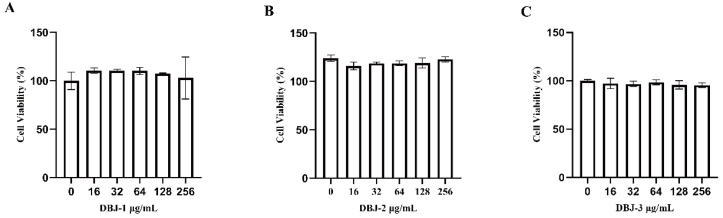
The effects of different DBJ extracts on AML-12 cell survival were evaluated after 24 h. (**A**) DBJ-1; (**B**) DBJ-2; (**C**) DBJ-3. All the data are presented as the means ± SDs (*n* = 6).

AML-12 cells were treated with different concentrations of DBJ extracts (16, 32, 64, 128, and 256 μg/mL) for 24 h (Figure 3A–C). Increasing the concentration of extracts did not affect cell viability, indicating that DBJ extracts are safe for AML-12 cells within the tested range of 16–256 μg/mL.

#### 3.3.2. Effect of Alcohol Concentration on the Viability of AML-12 Cells

**Figure 4:**
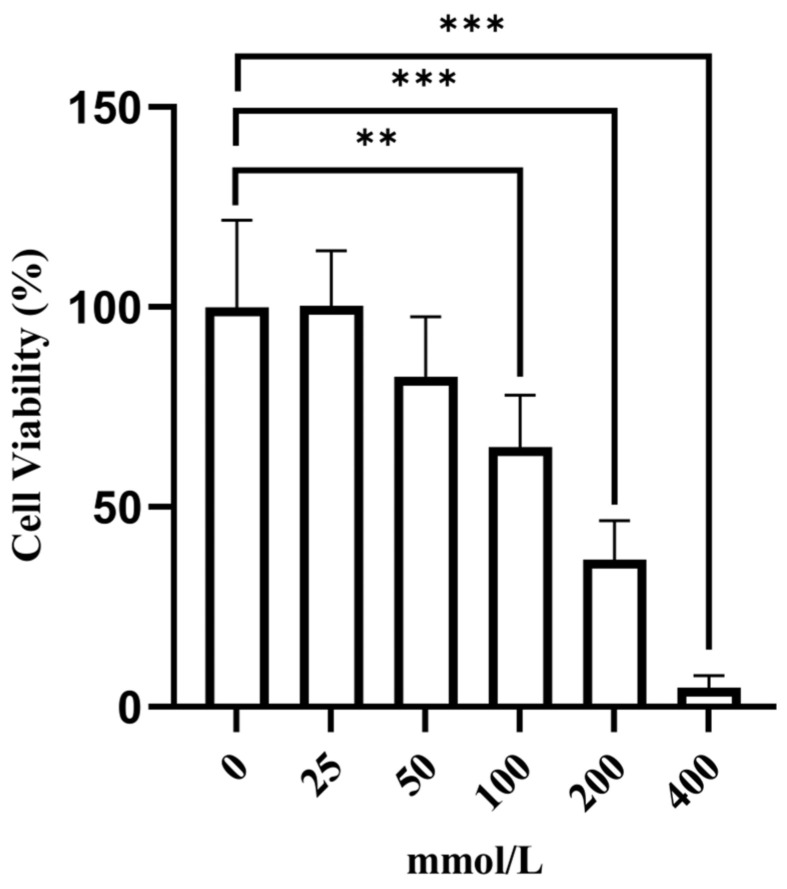
After 24 h, the survival rate of the AML-12 cells was determined with different concentrations of ethanol. All the data are presented as the means ±SDs (*n* = 6); ** *p* < 0.01 and *** *p* < 0.001 vs. the normal control group.

The cell viability decreased as the alcohol concentration increased, as shown in Figure 4. Compared with that of the normal control group, the cell viability of the 100 mmol/L alcohol group was approximately 60%. Thus, 100 mmol/L alcohol was selected for subsequent studies.

#### 3.3.3. Effect of DBJ Extract on the Viability of AML-12 Cells in the Presence of 100 mmol/L Ethanol

**Figure 5:**
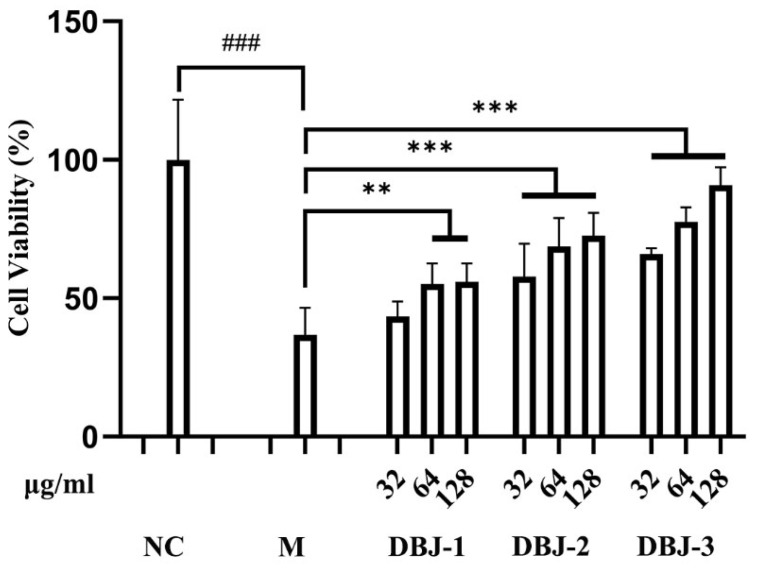
Alterations in the survival rate of AML-12 cells. All the data are presented as the means ± SDs (*n* = 6); ^###^ *p* < 0.001 vs. the NC group; ** *p* < 0.01 and *** *p* < 0.001 vs. the M group.

The effects of different DBJ extracts (32, 64, and 128 μg/mL) on the relative viability of AML-12 cells at a concentration of 100 mmol/L ethanol are shown in Figure 5. Compared with the NC group, the M group presented significantly lower cell viability (*p* < 0.001). In contrast, the treatment groups presented considerably greater cell viability than the M group. Among the different samples at the same concentration, DBJ-3 resulted in the highest cell viability, indicating a dose-dependent effect. These results suggested that within the concentration range of 32 to 128 μg/mL, different DBJ extracts effectively mitigate alcohol damage to AML-12 cells, with DBJ-3 showing the most prominent protective effect (*p* < 0.001).

### 3.4. Effect of DBJ-3 on Serum Biochemical Factors in Mice with Alcohol-Induced Liver Injury

**Figure 6:**
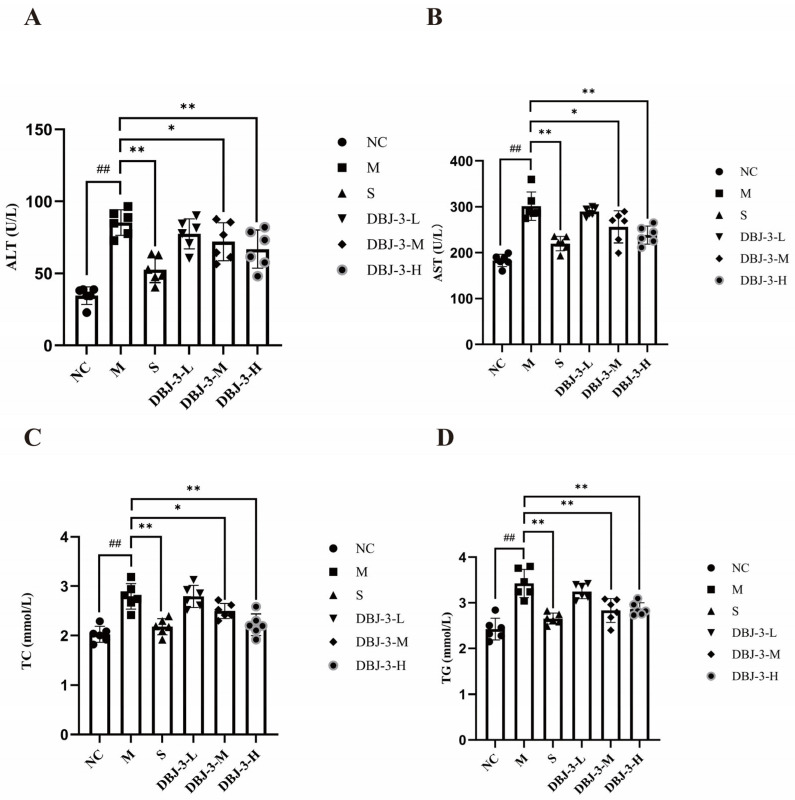
Changes in the serum biochemical indices. The NC group (normal control, distilled water), M group (model, 56% alcohol at 15 mL/kg), S group (silymarin, 60 mg/kg), DBJ-3-L group (100 mg/kg DBJ-3), DBJ-3-M group (300 mg/kg DBJ-3), DBJ-3-H group (600 mg/kg DBJ-3). (**A**) ALT; (**B**) AST; (**C**) TC; (**D**) TG. The symbol corresponding to each group represents the data for that group, and all the data are presented as the means ± SDs (n = 6); ^##^ *p* < 0.01 vs. the NC group; * *p* < 0.05 and ** *p* < 0.01 vs. the M group.

The effects of DBJ-3 on the serum ALT, AST, TC, and TG levels in AALI mice are shown in Figure 6. In the M group, the levels of these indicators increased significantly relative to those in the blank group (*p* < 0.01). The ALT, AST, TC, and TG levels in the S group were significantly lower than those in the model group (*p* < 0.01). The DBJ-3-H group presented significantly lower levels of ALT, AST, TC, and TG (*p* < 0.01). These results indicate that DBJ-3 can improve liver function indicators and protect against AALI.

### 3.5. Expression Levels of Alcohol Metabolism-Related Enzymes

**Figure 7:**
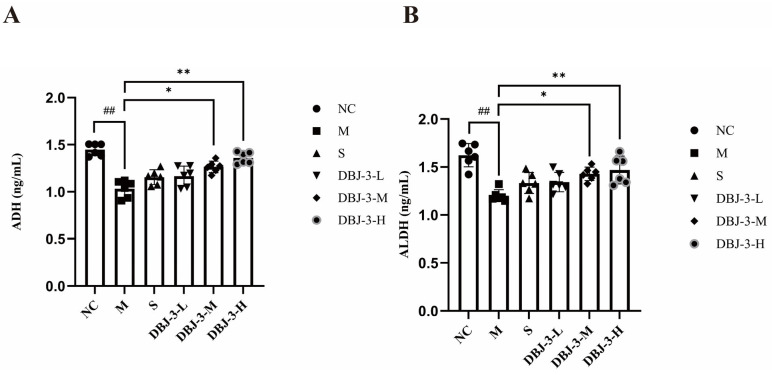
Changes in ADH and ALDH levels in mouse liver tissue. (**A**) ADH; (**B**) ALDH. All the data are presented as the means ± SDs (*n* = 6); ^##^ *p* < 0.01 vs. the NC group; * *p* < 0.05 and ** *p* < 0.01 vs. the M group.

The effects of DBJ-3 on hepatic ADH and ALDH levels in AALI mice are shown in Figure 7. The ADH and ALDH levels in the model group were significantly lower than those in the normal control group (*p* < 0.01). Compared with those in the model group, both the ADH and ALDH levels significantly increased in the DBJ-3-H groups (*p* < 0.01), increasing alcohol-metabolizing enzyme activities while accelerating alcohol metabolism.

### 3.6. Histopathological Changes

**Figure 8:**
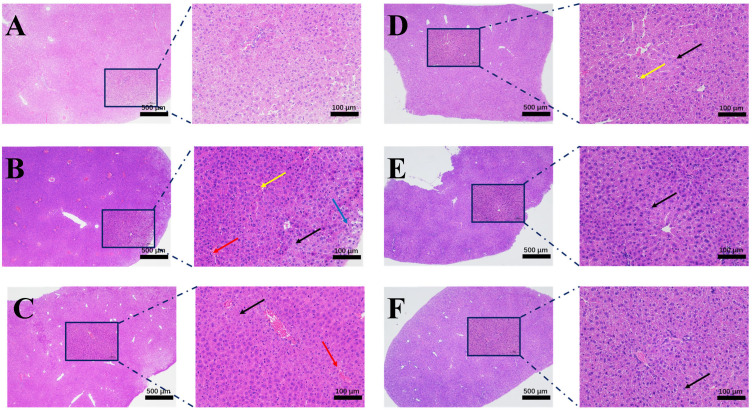
Histopathological sections from each group. (**A**) NC group; (**B**) M group; (**C**) S group; (**D**) DBJ-3-L group; (**E**) DBJ-3-M group; (**F**) DBJ-3-H group. The left image scale bar was 500 μm, and the right image scale bar was 100 μm in each group. Yellow arrows indicate cellular swelling and cytoplasmic looseness with light staining, blue arrows indicate lipid vacuolation in hepatocytes, and red arrows indicate a small amount of vascular congestion. The black arrows represent slight hydropic degeneration of many hepatocytes in the liver tissue.

Histopathological changes in the mice from the different experimental groups were assessed by staining the tissues with hematoxylin and eosin (H&E) (Figure 8). The NC group (Figure 8A) presented a normal liver structure with an intact cytoplasm, prominent nuclei, clear venous vessels, and well-defined cell boundaries without steatosis or inflammatory infiltration. In contrast, as indicated by the four arrows, group M (Figure 8B) exhibited hepatocyte cell swelling, lipid vacuolation in hepatocytes, a minor degree of vascular congestion, and degeneration of hepatocytes. Compared with the M group, the S group (Figure 8C) presented a minor degree of vascular congestion, and slight hydropic degeneration. Furthermore, liver damage in the DBJ-3-L, DBJ-3-M, and DBJ-3-H groups (Figure 8D–F) was significantly decreased, leading to a reduction in cell swelling and cellular lipid vacuolization. Notably, the DBJ-3-H group displayed only a slight degree of adipocyte degeneration, indicating a dose-dependent hepatoprotective effect.

### 3.7. Regulatory Effects of DBJ-3 on ROS and Antioxidant Parameters in Liver Tissue

**Figure 9:**
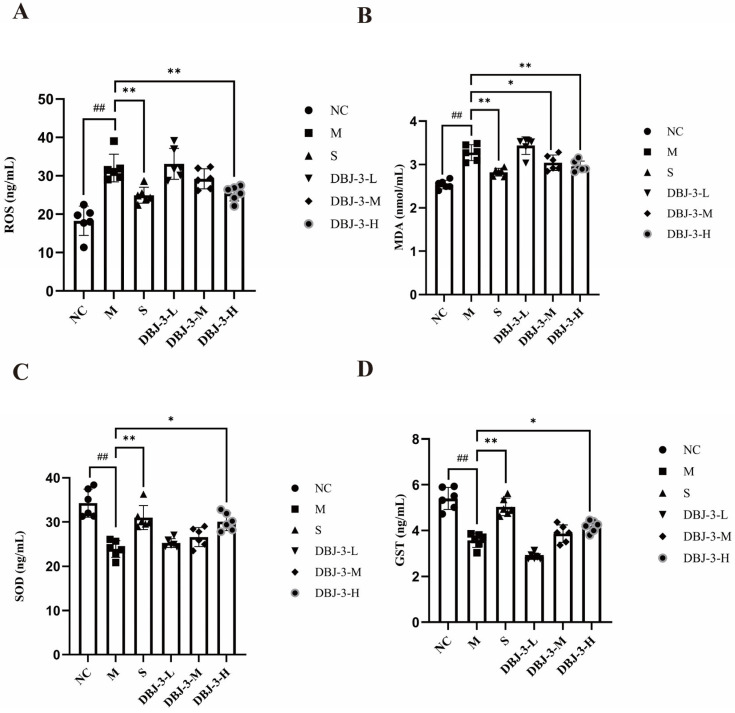
Changes in ROS, MDA, SOD, and GST levels in mouse liver tissues. (**A**) ROS; (**B**) MDA; (**C**) SOD; (**D**) GST. All the data are presented as the means ±SDs (*n* = 6); ^##^ *p* < 0.01 vs. the NC group; * *p* < 0.05 and ** *p* < 0.01 vs. the M group.

The effect of DBJ-3 on ROS levels in hepatic homogenates from AALI mice is shown in Figure 9A. Compared with the blank group, the model group presented significantly higher ROS levels (*p* < 0.01). The ROS levels in hepatic homogenates from the S and DBJ-3-H groups were significantly lower than those in the M group (*p* < 0.01), indicating that DBJ-3 may promote ROS clearance in vivo to relieve alcohol-induced oxidative stress.

The effects of DBJ-3 on the SOD, MDA, and GST levels in hepatic homogenates from AALI mice are presented in Figure 8B–D. Compared with those in the NC group, the SOD and GST contents in the M group decreased significantly (*p* < 0.01), whereas the MDA content increased significantly (*p* < 0.01). Both the S and DBJ-3-H groups presented significantly higher GST and SOD levels in hepatic homogenates (*p* < 0.05), indicating an increased antioxidant capacity in vivo. Additionally, both the S and DBJ-3-H groups presented considerably lower MDA levels (*p* < 0.01), alleviating lipid peroxidation in the mice.

### 3.8. Inflammatory Cytokine Levels in Liver Tissues

**Figure 10:**
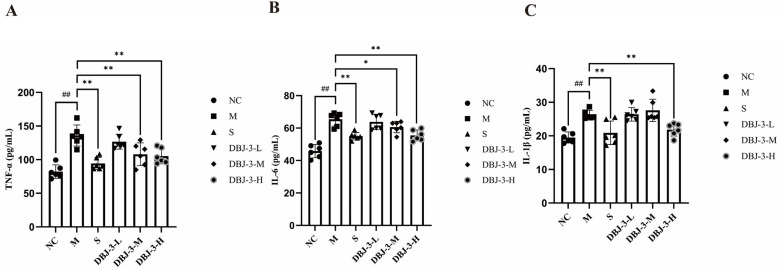
Changes in TNF-α, IL-1β, and IL-6 levels in mouse liver tissues. (**A**) TNF-α; (**B**) IL-6; (**C**) IL-1β. All the data are presented as the means ± SDs (*n* = 6); ^##^ *p* < 0.01 vs. the NC group; * *p* < 0.05 and ** *p* < 0.01 vs. the M group.

The preventive effects of DBJ-3 on tumor necrosis factor (TNF-α), interleukin-6 (IL-6), and interleukin-1β (IL-1β) levels in hepatic homogenates from AALI mice are illustrated in Figure 10. The levels of these three cytokines were significantly greater in the M group than in the NC group (*p* < 0.01). The levels of the three cytokines were significantly lower in both the S and DBJ-3-H groups than in the M group (*p* < 0.01), indicating that the levels of inflammatory factors were considerably lower in the S and DBJ-3-H groups than in the M group, thus alleviating the inflammatory response.

### 3.9. Regulation of the ADH1B/ALDH2 Signaling Pathway by DBJ-3

**Figure 11:**
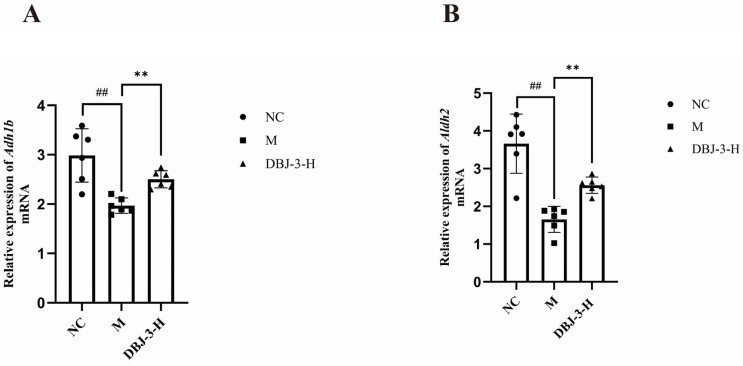
Expression levels of the *Adh1b* and *Aldh2* mRNAs. (**A**) *Adh1b*; (**B**) *Aldh2*. All the data are presented as the means ± SDs (*n* = 6); ^##^ *p* < 0.01 vs. the NC group; ** *p* < 0.01 vs. the M group.

As shown in Figure 11, *Adh1*b and *Aldh2* expression was significantly lower in the M group than in the NC group (*p* < 0.01). In contrast, *Adh1*b and *Aldh2* expression in the DBJ-3-H group was significantly greater than that in the M group (*p* < 0.01), indicating that DBJ-3-H plays a role in enhancing *Adh1b* and *Aldh*2 expression, thereby improving alcohol metabolism in mice.

**Figure 12:**
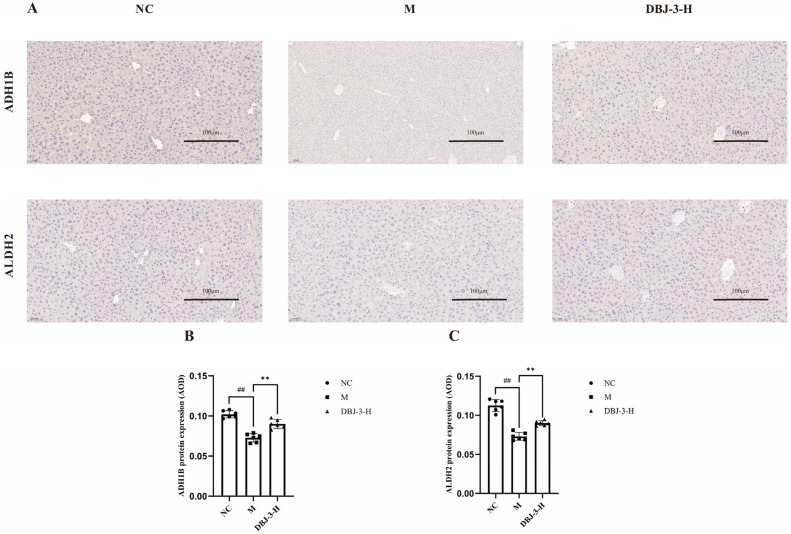
Immunohistochemical staining analysis of ADH1B and ALDH2 in the different groups. (**A**) Images illustrating the immunoreactivity of ADH1B and ALDH2; (**B**) ADH1B; (**C**) ALDH2. All the data are presented as the means ±SDs (*n* = 6); ^##^ *p* < 0.01 vs. the NC group; ** *p* < 0.01 vs. the M group.

Protein expression in liver tissue sections embedded in paraffin was analyzed using immunohistochemistry (IHC). A brownish yellow color was considered positive (Figure 12A). The integrated optical density (IOD) ratio was calculated to determine the average optical density (AOD), facilitating relative protein quantification and analysis of the IHC results on the basis of the AOD [57]. The findings of IHC for ADH1B and ALDH2 in liver tissues are illustrated in Figure 12B,C. ADH1B and ALDH2 protein expression was significantly lower in the M group than in the NC group (*p* < 0.001), whereas their expression levels were greater in the DBJ-3-H group (*p* < 0.001). Figure 12A shows that the color development in the M group was less pronounced than that in the NC group. Furthermore, compared with that in the M group, color development in the DBJ-3-H group was greater for ADH1B and ALDH2, indicating that DBJ-3-H activated the ADH1B and ALDH2 pathways, thereby increasing alcohol metabolism in the body. These results further validated the RT-qPCR findings.

### 3.10. Role of the NF-κB Pathway in Gene Expression

**Figure 13:**
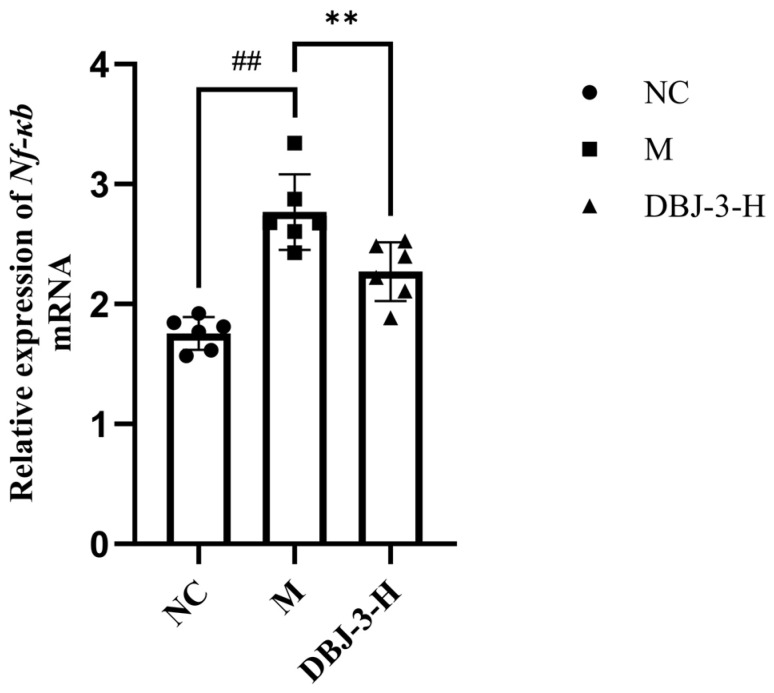
*Nf-κb* mRNA expression. All the data are presented as the means ± SDs (*n* = 6); ^##^ *p* < 0.01 vs. the NC group; ** *p* < 0.01 vs. the M group.

As shown in Figure 13, *Nf-κb* expression in mouse liver tissues was significantly greater in the M group than in the NC group (*p* < 0.01). Conversely, *Nf-κb* expression in the DBJ-3-H group was significantly lower than that in the M group (*p* < 0.01). Therefore, DBJ-3-H inhibits *Nf-κb*, leading to anti-inflammatory effects and mitigating the inflammatory response.

**Figure 14:**
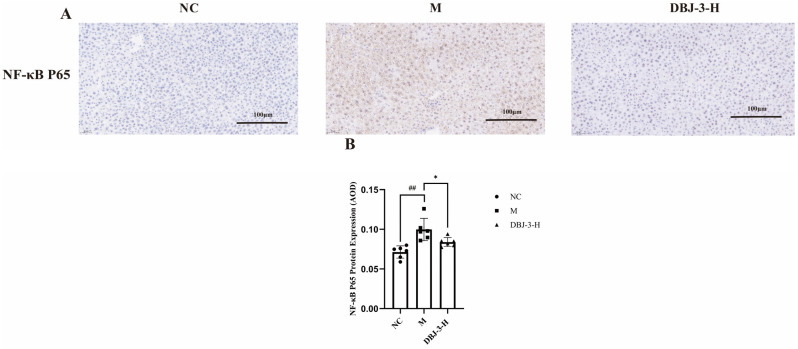
IHC analysis of NF-κB in the different groups. (**A**) Images illustrating the IHC of NF-κB; (**B**) NF-κB P65 protein expression. All the data are presented as the means ± SDs (*n* = 6); ^##^ *p* < 0.01 vs. the NC group; * *p* < 0.05 vs. the M group.

The results of the IHC analysis of NF-κB in liver tissue sections are shown in Figure 14. As depicted in Figure 14B, NF-κB protein levels were significantly greater in the model group than in the blank group (*p* < 0.01). In contrast, NF-κB protein levels were significantly lower in the DBJ-3-H group (*p* < 0.05). As illustrated in Figure 14A, the M group presented a darker brown color, whereas the DBJ-3-H group presented reduced color development. This observation suggests that DBJ-3-H acts as an inhibitor of the NF-κB pathway. These changes can enhance anti-inflammatory ability in mice, which is consistent with the RT-qPCR results.

## 4. Discussion

Ethnic medicine is a crucial element of China’s medical heritage and scientific practice. Originating from ethnic minority regions, it often remains underappreciated outside these areas. However, the influence of ethnic medicine on treating local diseases and promoting overall community health, coupled with the knowledge passed down through generations, highlights its significant value. This value deserves further exploration and application. Dai Bai Jie, a widely used Dai medicine, has been employed for an extended period in various Dai communities, both domestically and internationally [58]. However, the original plant source of Dai Bai Jie has only been identified recently. Unfortunately, the scientific name of the base plant of Dai Bai Jie has not been adequately emphasized [59]. Through experimental research, the original plant of Dai Bai Jie was determined to be *Marsdenia tenacissima* (*Roxb.*) *Moon*, rather than *Dregea sinensis Hemsl* [60].

The literature suggests that Dai Bai Jie is rich in saponins [61,62,63]. Saponins are typically separated through boiling in water [64], methanol [65], ethanol [66], or butanol [67], and various extraction methods can significantly influence the total saponin content in Dai Bai Jie. The total saponin content determination kit employed in this study is based on the vanillin–perchloric acid colorimetric method [33,68]. The ginsenoside Re included in the kit serves as the standard for constructing the standard curve. Ginsenoside, a representative of total saponins, possesses a relatively stable structure and properties, facilitating quantitative analysis [69,70,71]. Owing to the lack of a clearly defined chemical composition for Dai Bai Jie saponins, we utilized a total saponin content determination kit to assess and compare the total saponin content across various extraction methods. Our findings indicate that the extract obtained from Dai Bai Jie using 95% alcohol presented the highest total saponin content.

As a mouse liver cell line, AML-12 cells possess certain hepatocyte characteristics and metabolic functions, although they cannot fully replicate the complex structure and function of liver tissue [44,72,73]. By assessing the viability of AML-12 cells, we first confirmed that the three extracts of Dai Bai Jie exhibited no cytotoxicity. We subsequently determined that the optimal ethanol concentration in the medium was 100 mmol/L. When the ethanol concentration was 100 mmol/L, we observed and compared the preventive effects of the three extracts on AML-12 cells. The results demonstrated that the relative activity of AML-12 cells was highest when they were treated with the 95% ethanol extract of Dai Bai Jie, and it had a more significant protective effect on AML-12 cells than the water extract or the 50% ethanol extract.

The use of traditional Chinese medicine for treating AALI is increasing [74,75,76,77]. Notably, reports in the literature indicate the potential benefits of analogous saponins in the prevention and treatment of AALI. [23,78,79,80]. For example, the ginsenoside Rk2 (Rk2) alleviates alcohol-induced hepatic inflammation by inhibiting NLRP3 inflammasome signaling in the liver and improving alcohol-induced intestinal barrier dysfunction by promoting the gut NLRP6 inflammasome [78]. Platycodin D (PD), extracted from the aerial parts of *Platycodon grandiflorus*, protects against alcohol-induced hepatocyte apoptosis and steatosis when administered as a pretreatment [79]. Escin reduces hepatic inflammatory mediators, mitigates inflammatory responses, upregulates the expression of 11β-hydroxysteroid dehydrogenase 2 and glucocorticoid receptors, and enhances endogenous antioxidant capacity, thereby alleviating liver injury induced by endotoxemia [81]. In our experiments, DBJ-3 exhibited the highest saponin content, and we identified eight saponins that share structural similarities with those reported in the literature, as determined by UPLC-MS analysis. Excessive alcohol consumption poses significant health risks. The primary approach to mitigate these risks involves reducing alcohol levels in the body [82]. Upon ingestion, alcohol is quickly absorbed in the gastrointestinal tract and metabolized primarily to acetaldehyde by ADH, with additional metabolism via the cytochrome P450 2E1 system in the smooth endoplasmic reticulum, which is particularly pronounced in cases of alcoholic liver damage. A portion of alcohol is also metabolized by CAT to generate acetaldehyde [4]. Acetaldehyde is further metabolized to acetate by ALDH [83,84]. Enhancing genes and proteins involved in alcohol metabolism, reducing alcohol and acetaldehyde levels in the body, and limiting alcohol intake or absorption are direct strategies to minimize associated risks [82]. ADH and ALDH are the primary enzymes involved in the pathway that converts ethanol to acetate [85,86]. ADH, a cytoplasmic enzyme encoded by seven genes (ADH1–ADH7), includes ADH1B, which has a lower Km and higher activity in converting ethanol to acetaldehyde than the other six ADH subtypes do. ALDH2 is a mitochondrial enzyme with a low Km for acetaldehyde (<5 mM) and plays a key role in acetaldehyde metabolism [87,88,89].

In this study, the preventive administration of DBJ-3 significantly increased ADH and ALDH levels in mouse livers. These findings suggest that DBJ-3 enhances ADH and ALDH synthesis, facilitating alcohol breakdown in the body. This process helps mitigate the adverse effects of alcohol abuse and acetaldehyde accumulation, which are evident in liver tissue sections showing cell damage and hepatic sinusoidal congestion. Prophylactic DBJ-3 treatment considerably improved these symptoms and modulated liver function indicators. The upregulation of ADH1B and ALDH2 expression indicates that DBJ-3 may increase alcohol metabolism via the ADH1B/ALDH2 pathway. The unique ability of DBJ-3 to accelerate alcohol metabolism in mice accelerates the metabolism of ethanol and acetaldehyde, as observed in histological analyses showing improved liver health.

The levels of AST, ALT, and TC are commonly used as serum biomarkers for the early detection of liver injury [90,91]. AST and ALT levels reflect liver function and indicate the extent of hepatocyte necrosis. Liver damage can disrupt cholesterol synthesis and metabolism, leading to elevated serum TC levels [14,92]. TG serves as a crucial indicator of hepatic steatosis, with excessive alcohol intake disrupting lipid metabolism and causing TG accumulation [78,93,94]. After alcohol-induced injury, the levels of AST, ALT, TC, and TG were significantly elevated in mouse serum. However, pretreatment with DBJ-3 notably reduced these biochemical markers, restoring metabolic function in the mice. These findings suggest that DBJ-3 can mitigate alcohol-induced liver cell damage and improve fat metabolism.

Moreover, AALI is closely related to the activities of ADH and ALDH, which directly influence the production and breakdown of acetaldehyde during ethanol metabolism. Elevated ADH activity and reduced ALDH activity can lead to the accumulation of acetaldehyde, a toxic compound known to induce oxidative stress. Moreover, acetaldehyde can trigger inflammation in conjunction with endotoxins produced during ethanol metabolism in the body. The results of the alcohol metabolism index revealed a significant decrease in ADH and ALDH levels in the liver damage model group, whereas the treatment group demonstrated a substantial increase in the levels of these enzymes. Furthermore, DBJ-3 was found to increase ADH1B and ALDH2 gene and protein expression in mice. These results indicate that DBJ-3 accelerates alcohol metabolism in mice by modulating the ADH1B/ALDH2 pathway, thus alleviating liver cell damage associated with excessive alcohol consumption.

Metabolizing large quantities of ethanol generates excessive ROS, which can disrupt the balance of crucial antioxidative enzymes such as SOD and GST in the liver. This oxidative stress leads to protein oxidation, lipid peroxidation, decreases antioxidant function, and impairs mitochondrial activity. Malondialdehyde (MDA) serves as a marker of lipid peroxidation, indicating the extent of oxidative damage and tissue injury caused by peroxidation [95]. Oxidative stress can lead to an increase in oxygen free radicals and other active oxide substances in the cell, which can activate the inflammatory response, causing the infiltration of inflammatory cells and the release of inflammatory mediators. The NF-κB signaling pathway and ROS have complex interactions. The transcription of NF-κB-dependent genes can influence ROS levels within cells, while ROS levels can also impact the activity levels of NF-κB. The NF-κB family of transcription factors consists of five members, RelA (p65), RelB, c-Rel, NF-κB1 (p105), and NF-κB2 (p100), which are further processed into p50 and p52, respectively. Activation of the NF-κB pathway primarily occurs through the stimulation of proinflammatory receptors, including those in the TNF receptor superfamily, the Toll-like receptor (TLR) family, and cytokine receptors for interleukins. Additionally, activation can be triggered by the toxic substance LPS. Oxidative stress can exacerbate inflammation in the body. Excessive ethanol intake increases intestinal permeability, allowing lipopolysaccharide (LPS) from the intestine to enter the liver. LPS binds to CD14 and TLR-4 receptors on the surface of Kupffer cells, activating them and triggering NF-κB activation. NF-κB induces the liver to produce proinflammatory factors, such as TNF-α, IL-1β, and IL-6, which intensify the inflammatory response [5,96]. DBJ-3 can reduce ROS levels, mitigate oxidative damage and MDA levels, improve lipid peroxidation, and increase the levels of antioxidant enzymes such as SOD, CAT, and GST. These findings suggest that DBJ-3 may alleviate oxidative stress by increasing the synthesis and activity of antioxidant enzymes or by reducing their degradation, thus increasing antioxidant levels in mouse livers. Following acute alcohol-induced injury, the NF-κB pathway is activated. DBJ-3 effectively regulates mRNA and protein expression in the livers of AALI mice by inhibiting NF-κB expression; therefore, DBJ-3 reduces the concentrations of TNF-α, IL-1β, and IL-6 in hepatocytes, indicating that it might have anti-inflammatory effects, leading to reduced inflammation.

## 5. Conclusions

In summary, DBJ-3 presented the highest saponin content. The composition of DBJ-3 was analyzed using UPLC-MS, leading to the identification of a total of eight saponins. Additionally, DBJ-3 has preventive and protective effects on AML-12 cells in an ethanol environment. DBJ-3 effectively mitigated AALI in mice through dual pathways. It enhances ADH1B/ALDH2 alcohol metabolism by increasing ADH and ALDH levels, aiding in alcohol breakdown. Additionally, DBJ-3 increased SOD, CAT, and GST antioxidant activity; reduced ROS and MDA levels; and mitigated lipid peroxidation. Additionally, DBJ-3 suppressed NF-κB gene and protein expression; decreased the levels of the inflammatory factors TNF-α, IL-6, and IL-1β; and alleviated inflammatory responses. The protective effects of DBJ-3 against ALD-induced damage are likely attributed to its combined influence on the ADH1B/ALDH2 and NF-κB pathways.

## Figures and Tables

**Table 1:** Information on the primers used in this study.

Gene	Species	Forward (5′–3′)	Reverse (3′–5′)
*Adh1b*	Mouse	CTGTGGGTTCTAACTGGCTATG	GGCAAACTTGTCCTTGTTGATGT
*Aldh2*	Mouse	TTCGGCAGGAGAATGTGTATGA	CGATTTGATGTAGCCGAGGATCT
*Nf-κb p65*	Mouse	CTTCTGGGCCTTATGTGGAGATC	GGTCCTGTGTAGCCATTGATCTT
*Gapdh*	Mouse	ACTCTTCCACCTTCGATGCC	TGGGATAGGGCCTCTCTTGC

**Table 2:** Standard solution concentration and absorbance.

Concentration (mg/mL)	Absorbance (ΔA)
0.1	0.2433
0.2	0.39452
0.3	0.54096
0.4	0.70112
0.5	0.84528
0.6	1.03262

**Table 3:** Saponins in DBJ-3.

Compounds	tR	Formula	Ionization Model	Exact Mass (Da)	Structures	Percentage of Relative Abundance (%)
2,3,23-Trihydroxyolean-12-en-28-oic acid	7.28	C30H48O5	[M-H]^−^	488.3502	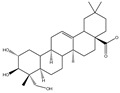	49.15
2-Aldehydo-A(1)-norlup-20(29)-en-27,28-dioic acid (Zizyberanal acid)	8.58	C30H44O5	[M-H]^−^	484.3189	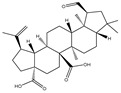	42.15
3β,19-Dihydroxyurs-12-en-28-oic acid	8.93	C30H48O4	[M-H]^−^	472.3553	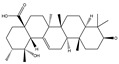	100
2α,3β-Dihydroxylup-20(29)-en-28-oic acid	9.30	C30H48O4	[M-H]^−^	472.3553	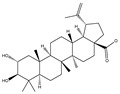	90.72
3β,25-Epoxy-3-hydroxyolean-18-en-28-oic acid	10.60	C30H46O4	[M-H]^−^	470.3396	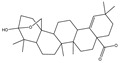	2.04
Oleanolic acid	11.00	C30H48O3	[M-H]^−^	456.3603	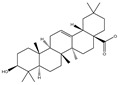	7.12
3-Oxolup-20(29)-en-28-oic acid	11.15	C30H46O3	[M-H]^−^	454.3447	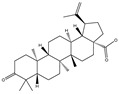	1.12
(3I(2),25R)-3-Hydroxy-24-methylene-9,19-cyclolanostan-26-oic acid	12.26	C31H50O3	[M-H]^−^	470.376	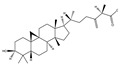	0.40

## Data Availability

Data are contained within the article.
